# High resolution real-time CMR of function and flow: initial clinical results

**DOI:** 10.1186/1532-429X-15-S1-E99

**Published:** 2013-01-30

**Authors:** Joachim Lotz, Jan M Sohns, Michael Steinmetz, Johannes T Kowallick, Christina Schulte, Wieland Staab, Arun Joseph, Klaus-Dietmar Merboldt, Dirk Voit, Shuo Zhang, Martin Uecker, Christina Unterberg-Buchwald, Gerd Hasenfus, Jens Frahm

**Affiliations:** 1 Diagnostic and Interventional Radiology, University Medical Center Goettingen, Goettingen, Germany; 2 Cardiology and Pulmonology, University Medical Center Goettingen, Goettingen, Germany; 3 Pediatric Cardiology, University Medical Center Goettingen, Goettingen, Germany; 4 NMR Forschungs gGmbH, Max Planck Institute Biophysical Chemistry, Goettingen, Germany

## Background

A new MRI technology for real-time MRI at high temporal and high spatial resolution was applied to CMR. First clinical applications cover dynamic imaging of wall motion and volume changes during cardiac arrhythmias as well as quantitative flow measurements under physiologic stress maneuvers.

## Methods

A recently introduced real-time MRI method based on undersampled radial FLASH sequences with image reconstruction by regularized nonlinear inversion was applied to CMR. Anatomical imaging in real time was performed at 34 ms temporal resolution (30 fps) using 1.5 mm in-plane resolution and 6 mm slice thickness. Real-time quantitative flow measurements employed two acquisitions at 20 ms resolution, yielding a temporal resolution of 40 ms (25 fps) at 1.3 mm in-plane resolution and 6 mm slice thickness. Healthy volunteers as well as patients with arrhythmia were examined in a clinical 3T MR scanner. The image series were analyzed using a modified standard software capable of dealing with 100 to 900 images per slice position. The ECG signal was co-registered for documentation and ease of image analysis.

## Results

The new high-resolution real-time MRI technique was used to analyze the beat-to-beat variability of patients with arrhythmia and to define ejection fractions in normal and arrhythmic episodes. Quantitative flow measurements were obtained in all major intrathoracic vessels during free breathing. Specific measurements during increased (Valsalva maneouver) and reduced intrathoracic pressure (Mueller maneouver) were obtained in healthy volunteers to document cardiovascular response to physiologic stressors. Regional wall motion, ventricular volumes, myocardial mass and ejection fraction were derived including standard deviations based on temporal variability of the heart cycle. Suitable software strategies for the analysis of the large datasets are indispensible to bring real-time CMR into clinical routine.

## Conclusions

Real-time CMR with high temporal and high spatial resolution emerges as a promising tool for future clinical studies.

## Funding

parts of the presented research was supported by the DZHK (Deutsches Zentrum für Herz-Kreislauf-Forschung) funded by BMBF, Germany.

